# Current state of geriatric services in Georgia: education, policy gaps, and recommendations

**DOI:** 10.3389/fpubh.2026.1742362

**Published:** 2026-02-12

**Authors:** Tamar Macharadze

**Affiliations:** 1 David Tvildiani Medical University, Tbilisi, Georgia; 2 Department Systems Physiology of Learning, Leibniz Institute for Neurobiology, Magdeburg, Germany; 3 Otto-von-Guericke Universitaet Magdeburg, Center for Behavioral Brain Sciences, Magdeburg, Germany

**Keywords:** geriatric anesthesia, geriatric education in Georgia, geriatric services, older adults, perioperative care, republic of Georgia, health system strengthening, geriatric policy

## Abstract

**Background:**

Georgia, an upper-middle-income country in the South Caucasus, is experiencing a rapid demographic shift toward an older population. Despite improvements in life expectancy and reductions in mortality, the healthcare system remains underprepared to address the complex medical and social needs of older adults.

**Methods:**

This mini-review synthesizes available information on geriatric education, workforce capacity, service provision, and the current state of geriatric research in Georgia. Findings are compared with neighboring countries and selected high-income European nations with established geriatric care systems, including hospital wards, outpatient clinics, and community-based programs.

**Results:**

Geriatric services in Georgia are limited. Medical education provides minimal exposure to geriatrics, postgraduate training is scarce, and dedicated hospital-based geriatric departments are largely absent. Nursing programs include basic geriatric modules, while short-term “Geriatric Assistant” courses offer practical skills for older adults care. Research focusing on older adults is sparse. In contrast, Germany, France, the Netherlands, and Nordic countries maintain well-structured, multidisciplinary hospital and community geriatric services supported by formal education, continuous professional development, and active research networks. Regional neighbors vary, with Turkey showing the most comprehensive system, and Armenia and Azerbaijan in early stages of development.

**Conclusion:**

Georgia urgently needs to integrate geriatrics into national health policy, strengthen workforce education, establish hospital and community geriatric services, and foster locally generated research. Learning from international models and aligning with frameworks such as the WHO Decade of Healthy Ageing can guide the development of a sustainable, patient-centered geriatric care system.

## Introduction

**Figure 1:**
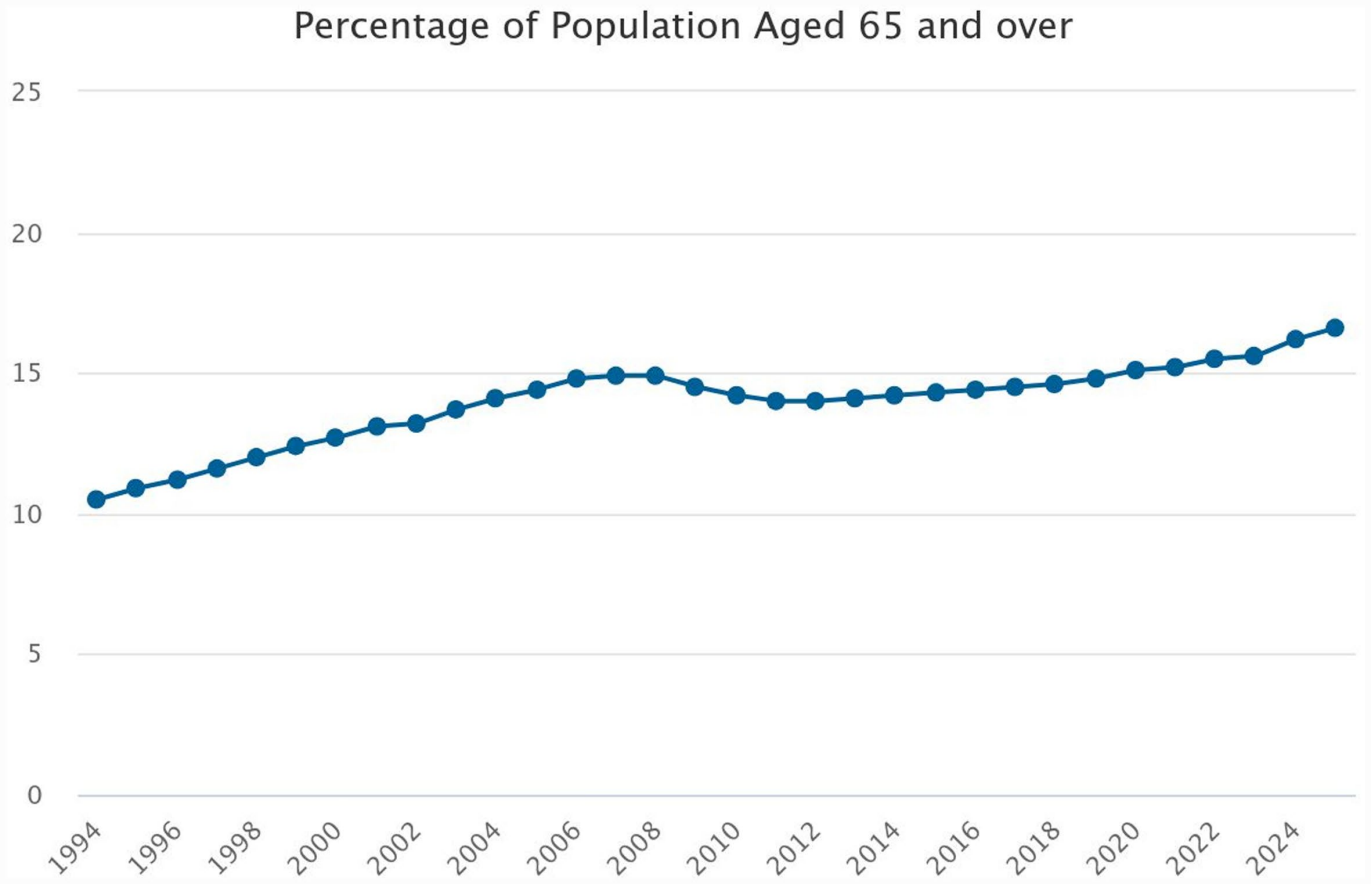
Trends in population aging in Georgia, showing the proportion of individuals aged 65 years and above. Data from the National Statistics Office of Georgia (Geostat) demonstrate a sustained increase in the older population.

Georgia, located in the South Caucasus, has an estimated population of 3.7 million, of which 16.2% are aged 65 years old or older [Figure 1; (1)]. Classified as an upper-middle-income country by the World Bank (2), Georgia is experiencing a rapid demographic shift toward an aging population, accompanied by increasing life expectancy (3). Our country has experienced notable improvements in life expectancy and declines in mortality rates in recent decades, largely due to enhanced management of noncommunicable diseases (NCDs), a reduction in fatal injuries, and advances in early disease detection and healthcare services (4). However, further efforts are needed to strengthen the quality of care and support systems for the growing older population. The healthcare system remains insufficiently equipped to address the complex medical and social needs of older adults. Comprehensive geriatric services—including dedicated hospital departments, outpatient clinics, and home-based rehabilitation programs—are absent, and there is a critical shortage of specialized geriatricians and trained nursing staff. The integration of geriatrics into national health strategies is minimal, and locally generated research on the health status, needs, and outcomes of older adults is scarce, limiting evidence-based planning for this vulnerable population.

To contextualize Georgia’s situation, this paper compares geriatric services in neighboring countries with those in selected high-income European countries. By comparison, countries such as Germany, France, the Netherlands, and the Nordic states have developed well-organized geriatric care systems, including hospital wards, outpatient clinics, and community-based programs. This brief review aims to present the current landscape of geriatric care in Georgia, identify gaps in policy and provide recommendations for strengthening services for its expanding older population.

### Organization and education of geriatric care in Georgia

The development of geriatric education and specialization in Georgia has undergone several structural changes over the past two decades. Several medical schools and universities have integrated geriatrics into their curricula, mainly within the framework of family medicine programs through courses worth just for 2 credits. However, geriatrics is not recognized as an independent discipline, and both teaching hours and clinical exposure remain limited. Between 2007 and 2014, “Gerontology–Geriatrics” was officially defined as a subspecialty of internal medicine under Order No. 136/n of the Minister of Labor, Health and Social Protection of Georgia (5). According to the State Certification Register department of the Ministry of Labor, Health and Social protection of Georgia, only two physicians are nowadays granted the right to independent medical practice in this field (6). Since 2015, the right to work in the field of clinical geriatrics within the specialty of internal medicine has been granted to individuals holding a certificate in the permitted specialty (Order No. 01-8/n of the Minister of Labor, Health and Social Protection of Georgia, “On Approval of Professional Competencies in Medical Specialties,” Appendix 37, Article 3) (7). Currently, there are no postgraduate or continuing medical education (CME) programs in geriatrics, and specialized hospital departments or outpatient services remain absent.

Around 30 educational institutions in the country offer long-term nursing programs that train competent general care nurses. As part of their curriculum, students complete a 3-credit course covering gerontology and the basic principles of geriatrics (8).

There is a training program called “Geriatric Assistant,” which consists of 144 h (approximately 6 weeks) and is offered by 14 professional education institutions in the country. During the course, participants acquire practical skills needed to care for older adults, including assistance with feeding, personal hygiene, dressing and undressing, and bed making.

Locally generated research on geriatry is extremely limited.

**Figure 2:**
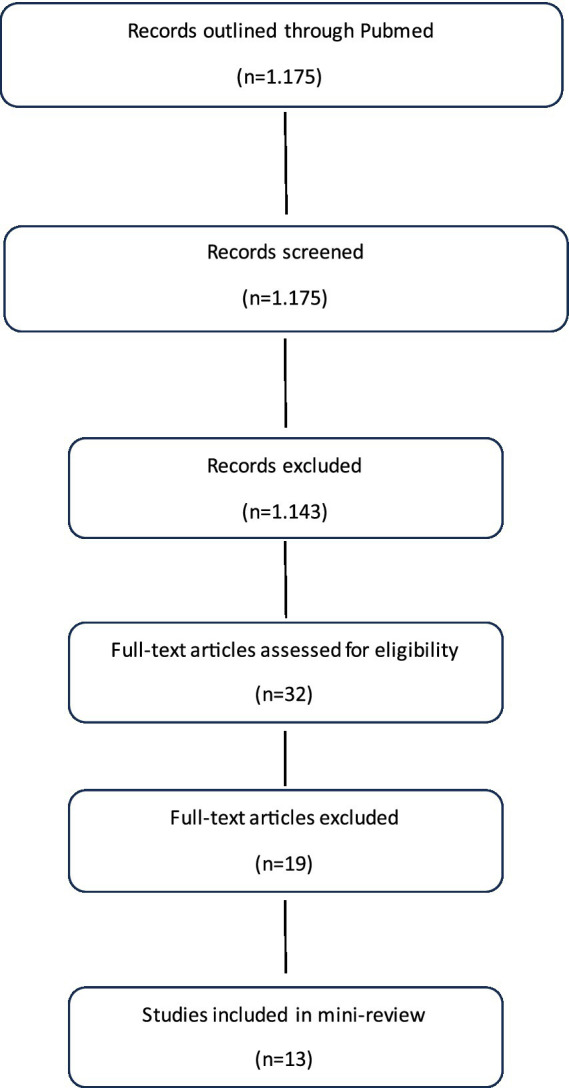
PRISMA flow diagram of PubMed search and study selection for geriatric health studies in Georgia.

We systematically searched **PubMed** for peer-reviewed studies on geriatric populations, aging-related conditions, healthcare services, and education in Georgia, published between 2010 and 2025, to summarize current evidence and identify gaps in research, education and public health for older adults in the country. Location terms (“Republic of Georgia”) were combined with geriatric keywords (“geriatrics,” “older adults,” “elderly,” “aging,” “frailty,” “cognitive disorders”) using **AND**, and synonyms with **OR**. After screening titles, abstracts, and full texts, 13 studies were included, covering oral health (9), fall-related trauma (10), social and ethical issues (11), loneliness (12), end-of-life care (13), gerontology research (14, 15), cancer outcomes (16), cognitive performance with HIV (17), dietary assessment (18, 19), preoperative frailty and postoperative cognitive disorders (20), mental health among internally displaced older adults (21), and education (Figure 2).

To date, no large-scale research studies or international conferences on geriatrics have been organized in the country. Our team has initiated research on older adults undergoing surgery with anesthesia, representing the first focused research initiatives in geriatric anesthesia in Georgia (20). In addition, we recently organized the first international conference (22) on cognitive disorders in Georgia, marking an important milestone in advancing geriatric research and raising awareness in the country, with the selected conference abstracts published in the Journal Anesthesia and Analgesia (23).

### Geriatric service in neighboring countries

To provide a regional perspective on geriatric care, we looked at neighboring countries, focusing on differences in service availability, workforce capacity, and professional training. As summarized in Table 1, Turkey has developed the most structured and integrated system, featuring dedicated hospital geriatric departments, outpatient clinics, and home-based rehabilitation programs, alongside formal geriatric subspecialty training and CME opportunities (24, 25). In Russia, while the geriatric workforce is more extensively trained and organized, service coverage is uneven across regions, and access to comprehensive geriatric care remains limited in certain areas (26). Armenia and Azerbaijan are still in the early stages of developing geriatric services. Both countries have very few hospital or outpatient geriatric departments, and workforce training is mostly limited to emerging educational initiatives for nurses and primary care providers (27–29). These disparities highlight the heterogeneity of approaches in the region and emphasize the need for strategic planning, capacity building, and targeted policy interventions. By comparing regional practices, we can derive valuable lessons for Georgia to inform the design and implementation of effective geriatric care services tailored to its demographic trends and healthcare infrastructure.

**Table 1:** Overview of geriatric services in selected neighbouring countries.

Country	Hospital geriatric departments	Outpatient clinics/day centres	Home-based/community programs	Number of geriatricians/specialists	Training and education	References
Turkey	Present in major hospitals	Present in some cities	Some home-based rehab programs	~109 across 41 centers	Subspecialty of Internal Medicine; fellowships & CME programs	Atmış et al. (24), Şimşek et al.(25)
Russia	Regional geriatric hospitals	1,303 outpatient geriatric rooms	Limited integration	~2,000 trained geriatricians	Varies by region; uneven coverage	Vlasova et al. (26)
Armenia	Rare/Very limited	Few community clinics	Caregiver school for nurses	Few trained specialists	Nursing courses, early-stage education	FAR USA (27)
Azerbaijan	Rare/Very limited	Very limited	Emerging pilot programs	Few trained specialists	Limited training opportunities	Physiology.az.(28), Science.gov.az.(29)

### Models and education of geriatric care in high-income countries

According to a recent scoping review by Masud et al. (30), high-income European countries demonstrate a more structured and proactive approach to geriatric education at the undergraduate level. Geriatrics is increasingly embedded in medical curricula through interdisciplinary courses, clinical rotations, and competency-based frameworks. This trend reflects a broader recognition of the importance of equipping future physicians with the skills to care for aging populations. Complementing these findings, the European undergraduate curriculum in geriatric medicine developed using an international modified Delphi technique (31) provides a harmonized educational framework that defines essential learning objectives and teaching methods. The same is with nurse education (32).

Postgraduate education in geriatric medicine is highly developed and well-structured across Europe. Many countries offer accredited specialization programs, fellowship opportunities, and international postgraduate courses supported by leading institutions such as the European Academy for Medicine of Ageing (EAMA) and the European Geriatric Medicine Society (EuGMS). In addition, several European research and training networks—such as the COST Action Program (European Cooperation in Science and Technology)—include dedicated initiatives focused on geriatric research, education, and capacity building, fostering collaboration between clinicians, educators, and scientists across member countries. These programs emphasize interdisciplinary competence, leadership in complex care, and lifelong learning through CME accredited by the European Accreditation Council for Continuing Medical Education (EACCME) (33–38).

In European countries, geriatric hospital care is highly structured and often organized around dedicated departments or wards that integrate acute, rehabilitation, and prehabilitation services (Table 2). In Germany, most university hospitals and large regional hospitals have dedicated gerontopsychiatry units, geriatric wards, offering comprehensive care, including early mobilization, preoperative optimization, nutritional support, and cognitive assessment for older adults prior to surgery (39). Prehabilitation programs are increasingly integrated into surgical pathways to reduce postoperative complications and functional decline (40, 41). France has developed specialized “Unités de Gériatrie” within general hospitals, often linked with outpatient geriatric clinics and home-based follow-up programs, emphasizing multidisciplinary care by geriatricians, nurses, physiotherapists, and social workers (42, 43). In the Netherlands, geriatric medicine is embedded in both university medical centers and general hospitals, with structured prehabilitation and rehabilitation pathways coordinated by multidisciplinary teams (44, 45). Similarly, the **Nordic countries** (Sweden, Denmark, Norway, Finland) implement national standards for geriatric wards and home-based programs, with strong emphasis on early functional assessment, prehabilitation prior to elective surgery, and community integration after discharge (46, 47). Across these countries, hospital-based geriatric services are closely linked with educational programs, research initiatives, and national guidelines, representing a mature model of integrated care for older adults.

**Table 2:** Overview of geriatric hospital care in high-income European countries.

Country	Type of service setting	Core service content	Coverage level	Integration/standartization	Quality indicators/outcomes	References
Germany	Dedicated geriatric wards and gerontopsychiatry units in university and regional hospitals	Comprehensive Geriatric Assessment (CGA), early mobilization, perioperative optimization, nutritional assessment, delirium prevention, cognitive screening	High in hospital-based care; outpatient follow-up varies by region	National guidelines; structured pathways; multidisciplinary teams	Reduced hospital stay, improved functional outcomes, lower readmission	Nau et al. (40), Fuchs et al. (41)
France	Specialized geriatric units (“Unités de Gériatrie”) within general hospitals, linked to outpatient clinics and home-care programs	Multidisciplinary geriatric care, functional and cognitive assessment, discharge planning, continuity of care	Nationwide framework with strong hospital–community linkage	National care pathways for older adults; structured coordination between inpatient and ambulatory care	Enhanced continuity of care; improved ADL (activities of daily living) scores	Assistance Publique – Hôpitaux de Paris (AP HP) (42), Everink et al. (43)
Netherlands	Geriatric medicine integrated in university and general hospitals, including acute geriatric community hospitals	Prehabilitation and rehabilitation pathways, CGA, multidisciplinary case management	High coverage with strong regional implementation	Nationally coordinated geriatric care pathways; outcome-oriented service organization	Improved post-op outcomes, reduced frailty, standardized assessments	Villars et al. (44), Ribbink et al. (45), Everink et al. (43)
Nordic countries	Hospital-based geriatric wards combined with community-based and home-care services	Early functional assessment, perioperative geriatric involvement, rehabilitation and community reintegration	Universal access within publicly funded healthcare systems	National clinical guidelines, population-level quality monitoring, strong primary–secondary care integration	High patient satisfaction; reduced hospital readmissions; robust frailty monitoring	Agerholm et al. (46), Agerholm et al. (47)

Table 2 highlights substantial differences in geriatric service organization, coverage, and integration across European countries. Germany and the Netherlands employ structured hospital-based geriatric pathways with standardized assessments, perioperative optimization, and multidisciplinary teams, ensuring consistent service quality. France emphasizes continuity of care between inpatient geriatric units, outpatient clinics, and home-based follow-up programs. The Nordic countries exemplify highly integrated models with national standards, universal access, and strong coordination between hospital and community services. These variations reflect differences in healthcare system organization, funding, and workforce capacity, which influence service accessibility, care continuity, and patient outcomes. By situating Georgia’s limited geriatric services alongside these models, the analysis underscores key gaps and identifies actionable areas for improvement, including workforce development, service standardization, and integration of community-based care.

### Key factors influencing senior citizen service development

Several factors explain why countries like Turkey have developed more advanced geriatric services compared with Georgia. Policy history plays a central role: In 2015 Turkey implemented the Health Transformation Program and the Healthy Aging Action Plan (2015–2020), creating structured strategies for geriatric care, service delivery, and monitoring population health outcomes (48, 49). These reforms included integrating geriatric modules into medical education and formalizing workforce competencies, which improved provider readiness and public trust (50, 51).

Fiscal investment has also been significant: dedicated funding for geriatric wards, community-based services, and rehabilitation programs enabled the rapid scaling of services and increased accessibility for older adults in Turkey (48). Additionally, cultural factors such as societal recognition of aging issues and advocacy by professional associations facilitated policy adoption and implementation (52).

Educational infrastructure further supports service development in Turkey. Master’s programs in gerontology and structured clinical training have strengthened the geriatric workforce, enhancing interdisciplinary care, evidence-based practice, and research capacity (49, 50). Research and social policy alignment complement these measures, ensuring that interventions address local needs while drawing on international best practices (53).

In contrast, Georgia lacks a comparable policy framework, dedicated fiscal allocation, and structured educational programs, resulting in limited hospital- and community-based services and sparse research output. The Turkish example illustrates how coordinated policy, investment, education, and cultural support can accelerate the development of comprehensive geriatric care systems.

## Discussion

Across Europe, the organization and maturity of geriatric services vary widely. Germany is often regarded as having the most comprehensive geriatric care system, with well-established hospital departments, early rehabilitation units, and national standards for interdisciplinary geriatric assessment and care. The Netherlands follows a highly integrated model that bridges hospital, nursing home, and community-based services, emphasizing functional independence and coordination between general practitioners and geriatricians. France maintains a strong hospital-based structure through specialized *Unités de Gériatrie*, linking inpatient, outpatient, and home-care services under coordinated management. In the Nordic countries—particularly Sweden, Denmark, and Finland—geriatrics is strongly community-oriented, with publicly funded, multidisciplinary teams focusing on home rehabilitation and long-term care continuity. These models collectively illustrate that sustained political commitment, national standards, and integration across care sectors are key determinants of successful geriatric service development.

When compared with neighboring countries, Georgia demonstrates a lower level of structured geriatric service development. Countries such as Turkey have introduced hospital-based geriatric units, outpatient geriatric clinics, and home-care services supported by national aging strategies and social care integration. In contrast, geriatric care in Georgia remains largely embedded within general internal medicine and emergency services, with limited availability of specialized geriatric or gerontopsychiatric units.

Armenia and Azerbaijan face similar constraints related to workforce shortages and limited specialization; however, Georgia’s rapidly aging population places additional strain on an already fragmented service structure. Unlike several Eastern European countries that have benefited from European Union–supported health system reforms, Georgia lacks a comprehensive national framework for geriatric care, resulting in uneven service availability and limited continuity of care.

In Georgia, geriatrics remains largely absent from national health policy and medical education despite growing ageing population. Geriatric research is very limited, and the scarcity of local data hinders evidence-based policy planning. Moreover, there is almost no dedicated infrastructure in hospitals to care for this vulnerable population. While some neighboring countries have made progress in establishing geriatric services, Georgia still lags behind and could benefit from adapting models from high-income countries.

To address the pressing gaps in geriatric care in Georgia, we propose a gradual, actionable strategy encompassing immediate-, intermediate-, and long-term aims, we recommend implementing institutions, and outline potential barriers along with strategies to address them. To ensure sustainable implementation of a nationwide geriatric care strategy in Georgia, a dedicated Geriatric Care Unit within the Ministry of Labour, Health and Social Protection of Georgia, led by a senior coordinator, should oversee policy development, standardization, and coordination across hospitals, primary care, and community services. Funding for this unit should come primarily from the Ministry of Labour, Health and Social Protection of Georgia, with supplementary support from regional authorities, international donors, public-private partnerships, and research grants. This structure will provide clear leadership, accountability, and resources for phased implementation, while facilitating integration of hospital- and community-based geriatric care.

### Immediate aims (1–2 years)

- To develop comprehensive training programs in Universities, medical schools, professional associations for doctors, nurses, and allied health professionals at all relevant medical establishments based on the best practices. This will support strengthening workforce capacity in geriatric care in the country. The development and implementation along with monitoring and evaluation should be led by the Ministry of Labour, Health and Social Protection of Georgia with support from academia, professional organisations and non-govermmental organisations (NGO).
- To develop and implement pilot community-based programs for frailty and cognitive screening. These efforts should be ideally be led by local and municipal health authorities in collaboration with primary care clinics and NGOs.
- To design and implement awareness raising campaigns on geriatrics for healthcare professionals and the public to help promote healthy aging and improve geriatric care. The lead agency here should be the Ministry of Labour, Health and Social Protection of Georgia supported by professional societies and NGOs.

### Intermediate aims (3–5 years)

To establish hospital-based geriatric units in tertiary centers and regional hospitals. This is critical for delivering coordinated, multidisciplinary care to older adults with complex medical, functional, and cognitive needs. By reducing complications, shortening hospital stays, and lowering readmissions, these units not only improve patient outcomes but also strengthen regional healthcare systems and advance geriatric education and research. Lead should be the Ministry of Labour, Health and Social Protection of Georgia and the effort will require strong support from hospital.

- To development and implement postgraduate training programs in geriatrics, including short-term fellowships and continuous professional development courses. These efforts should be led by higher education institution in collaboration with professional societies (e.g., European Geriatric Medicine Society (EuGMS)).
- To creation research networks focused on aging, geriatrics and quality of healthcare for older adults. This is essential for generating local evidence, monitoring the progress in this field and inform policy. The local academic institutions and research centers are best positioned to lead this initiative.
- To formalization of interdisciplinary care pathways for older adults in hospitals and establish a community care model. This will improve clinical outcomes, reduce complications and readmissions, and optimize resource use. Establishing linked community care models further supports smooth transitions from hospital to home, providing follow-up, rehabilitation, and social support that prevent functional decline and healthcare fragmentation. It should be led by hospitals and multidisciplinary clinical teams, coordinated by regional health authorities, and supported by primary care providers, community services, and academic institutions to ensure seamless, patient-centered care.

### Long-term aims (>5 years)

- To establish a nationwide system for geriatric care assessment in hospitals and primary care clinics to identify frailty, cognitive impairment, and other age-related conditions. This will ensure early intervention, coordinated care, and improved outcomes for older adults, facilitated through collaboration among the Ministry of Labour, Health and Social Protection of Georgia, regional authorities, hospitals, primary care providers, and geriatric specialists.
- To ensure continuous research on aging, geriatric care and related topics through dedicated research networks to foster generation of local knowledge and policy-relevant evidence.
- To align local policy with international best practices, frameworks, and guidelines such as, for example, the WHO Decade of Healthy Ageing, to guide national standards, funding priorities, and service development.

We recognize some of the proposed action items are ambitious and will require dedicated funding and professionals with necessary skills and experience. Additionally, we outline a set of potential barriers and provide some strategies to address them: 1. Lack of dedicated funding for establish geriatric care, services and education in Georgia. This can be addressed by everaging three phased implementation and seeking technical and financial support from national and international donor entities. 2. Workforce shortages: expand education and training programs while utilizing “train-the trainer” models. 3. Regional disparities: pilot interventions in diverse regions before national wide scaling.

By implementing this structured, context-specific approach, Georgia can develop a sustainable, patient-centered geriatric care system that addresses workforce, policy, and service gaps while promoting healthy aging.

### Main strengths and limitations

This mini-review provides a concise yet comprehensive overview of geriatric care in Georgia, synthesizing peer-reviewed evidence from 2010 onward. It highlights gaps in healthcare services, education, and policy, offering a foundation for actionable recommendations. By comparing Georgia with European and neighboring country models, the review contextualizes local challenges and identifies priorities for development. Its focused approach ensures relevance to public health, clinical practice, and future research planning.

This study has several limitations. First, our analysis is based on a narrative review of the available literature, and the number of peer-reviewed studies on geriatric care in Georgia remains limited. Second, heterogeneity in data sources and reporting across countries restricts direct comparisons of service coverage and quality indicators. Third, the absence of primary data collection or stakeholder interviews may limit insight into the actual delivery of geriatric services in practice.
